# Organization of a Hospital Ward Aimed at Admitting Patients with SARS-CoV-2: An Economic and Epidemiological Perspective

**DOI:** 10.3390/ijerph18189446

**Published:** 2021-09-07

**Authors:** Artur Z. Białoszewski, Dorota Gołąb-Bełtowicz, Monika Raulinajtys-Grzybek

**Affiliations:** 1 Department of Prevention of Environmental Hazards, Allergology and Immunology, Medical University of Warsaw, 02-097 Warsaw, Poland; artur.bialoszewski@wum.edu.pl; 2 Finance Department, Żeromski Specialist Hospital in Krakow, 31-913 Krakow, Poland; Dorota.beltowicz@gmail.com; 3 Department of Management Accounting, Warsaw School of Economics, 02-554 Warsaw, Poland

**Keywords:** SARS-CoV-2, COVID-19, pandemic, organization, infectious hospital ward, cost analysis, epidemiological situation, triage

## Abstract

The SARS-CoV-2 epidemic requires dynamic action on the part of the entire health care system to provide infected patients whose condition requires hospitalization with access to appropriate medical care and infrastructure, including oxygen devices and ventilators. The demand for specialized inpatient care has increased rapidly and in many areas exceeds the resources available to date. Individual hospitals must make investment and organizational decisions to increase their capacity to handle patients with SARS-CoV-2. The aim of the article is to present the organizational and investment steps taken to establish and maintain an infectious hospital ward as well as the clinical and financial consequences of this decision. The study was conducted in a hospital ward that was launched at the end of October 2020 to care for patients with SARS-CoV-2. A case study method was used. The department was characterized taking into account its importance for: (1) the regional level of health coverage of the population, (2) the organization of the hospital’s activities, (3) the financial and economic situation of the hospital.

## 1. Introduction

Coronavirus disease 2019 (COVID-19) is a severe acute respiratory syndrome resulting from a new type of coronavirus. The outbreak began in Wuhan, China, and soon was declared a pandemic by the World Health Organization on 11 March 2020 [1]. COVID-19 has affected most countries around the world. For most of the European countries it was the first serious epidemic in many years that threatened the life and health of citizens on a massive scale. In Poland, the first case of COVID-19 was reported on 4th of March in Lubuskie voivodship. The first peak of the epidemic occurred on 25 November 2020 (440 thousand active cases) and the second peak on 2 April 2021 (421 thousand active cases) [2].

The healthcare system had to quickly change its organization to meet the needs of constantly increasing numbers of positive patients, considering their frequent hospitalizations as well as intensive care needs. Such a change concerns many aspects of the functioning of healthcare institutions, mainly hospitals, including investment, organizational and financial aspects. 

Decisions on the organization of patient admissions require proper preparation of infrastructure facilities. One of the solutions implemented in several regions of the world are temporary hospitals [3]. Capacity of general wards is not sufficient during an epidemic, which requires adaptive measures to increase it abruptly. Temporary specialty hospitals expand the admission capacities while reducing the number of patients in other hospitals. They also centralize the flow of patients in the region which may help in controlling the epidemic. The construction of a temporary hospital is a huge investment challenge, associated with the use of appropriate technologies and approach to its implementation. Seventeen COVID-19 field hospitals were built in Wuhan. Their organization involved architects, engineers and infectious disease experts who, using the available infrastructure of stadiums, conference centers, and classrooms, transformed them into temporary hospitals. The process included determination of the best locations for patient beds, organization of the care process, fire escape pathways, water and electricity sourcing [4,5].

Most patients hospitalized with COVID-19 are admitted to general hospitals, where they are hospitalized either in infectious units or intensive care units, depending on their condition. The first point of contact for patients is the emergency room. The use of existing hospital infrastructure requires less investment than construction of a temporary hospital, however, as the authors have pointed out, it involves necessary organizational changes that shape the possibility of ensuring continuity of care. The authors of [6] also point out the importance of close collaboration between the emergency and other departments as well as the need for urgent transformation of other wards to sub-intensive infectious units for COVID-19 patients.

Important parts of the process include sustaining the strict triage of patients as well as organizing observation wards, which have been mentioned by the majority of respondents in a global Spanish study of EDs [7,8]. The most important elements of the triage procedure in case of the epidemic are free online consultations, differentiation of regions and pathways for patients in different conditions and levels of contagiousness, as well as giving priority to EDs in terms of material supply as well as access to other service areas in the hospital [7,9,10,11]. 

Flexibility in allocation of the workforce in order to maintain staffing levels in critical areas plays an important role in the crisis management process, especially in managing the hospital’s activities in the event of a pandemic. Some academics recommend implementation of mixed teams from different departments in order to accelerate the transfer of different skills [6]. It is also crucial to provide staff with personal protection equipment as well as the knowledge of how to use it properly [12]. 

Proper organization of healthcare should also minimize healthcare-associated infections. Factors limiting the number of infections are implementation of single-patient rooms and easily accessible hand sanitizer dispensers located near the patient’s bed [13]. Most hospitals arrange dedicated units for patients with known or suspected COVID-19, as well as taking other steps such as daily checking of staff members’ health and keeping staff home if sick or exposed [14]. 

Pandemics have considerable consequences on the total cost of the healthcare system [15]. However, it should be mentioned that, unless reimbursement equals expenses (which is rarely the case in Europe), the risk of covering additional costs is split between the payers and the provider. In Poland reimbursement for procedures is fixed (usually case-based) regardless of the actual expenses. Therefore, reorganization of a hospital also has consequences for its financial situation. On the one hand, the hospital incurs additional expenses related to the personnel involved in care, services, materials, supplies and renovation of existing space. These costs should be reflected in the corresponding revenues for the care of COVID-19 patients, calculated in accordance with the reimbursement system of a country. On the other hand, the consequences of the epidemic and the need to prioritize COVID-19 patients with dynamically changing forecasts for the development of the situation is a limitation on the remaining operations of the hospital and can result in lost revenue for other services [16].

The abovementioned studies indicated various aspects of hospital organization during the epidemic. Most of them focused on one of the following areas: investment and construction, process and organization, epidemiology or finance. The aim of this study is to present a case study of a hospital operating in southern Poland during the so-called second wave of the epidemic, which reached its peak in Poland in October and November 2020. Changes in the way the hospital was organized are presented from an epidemiological perspective, indicating the importance of the hospital in the regional security network. It also illustrates the organizational and investment measures that were implemented to ensure patient care, and a calculation of the financial implications of the steps taken. 

## 2. Materials and Methods

A case study was carried out in one of the hospitals located in southern Poland, in the Małopolska region. During the second wave of the epidemic, a new department dedicated to the treatment of COVID-19 was established in the hospital, which allowed for the isolation of analysis regarding the treatment of patients with COVID-19 from the remaining activity of the hospital. 

We undertook a retrospective cost study over a two-month period (23 October–31 December) of the operation of the COVID-19 Department. The analysis covered data available in the hospital, including data on the treatment process of patients, financial data, human resources and organizational data, including the triage procedure and epidemiological safety procedures. 

The financial data for the analyzed period was obtained from the hospital’s financial systems. Lost revenues and variable cost savings due to fewer patients in the Surgery and Orthopedic Surgery Departments were estimated based on data from comparative periods. The analysis did not take into account the indirect (social) costs resulting from the limited availability of surgical and orthopedic services and the delays in the treatment process for patients. These costs are outside the scope of this analysis; however, they are significant from the patients’ perspective.

Hospital data was presented against the epidemiological data on the course of the epidemic in the region. Data was collected on the basis of reports provided by the Ministry of Health, data from the WSEZ, PSSE, Voivodship Offices, and data obtained via requests for access to public information.

## 3. Epidemiological Situation in the Region

**Figure 1:**
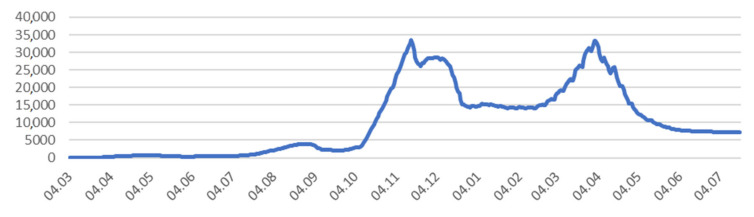
Number of active cases (COVID-19) in Małopolska. Source: Data collected on the basis of reports provided by the Ministry of Health, data from the WSEZ, PSSE, Voivodship Offices, and data obtained via requests for access to public information.

Lesser Poland Voivodeship, also known as Małopolska, is a province in southern Poland. It has an area of 15,108 square km (5833 sq mi), and a population of 3,404,863 (2019). The province is divided into 22 counties: 3 city counties and 19 land counties [17]. Counties of Małopolska are diversified not only in terms of socioeconomic conditions but also in terms of the number of cases and the structure of their sources. The first case (Figure 1) of COVID-19 in Małopolska was reported on 9 March 2020, shortly after the virus reached Poland [18]. 

Retrospective data revealed two waves of disease that hit the area. The first peak in of the epidemic in Małopolska occurred at a similar point to that of the whole country on 15 November 2020 (33.5 thousand active cases) and the second peak occurred on 1 April 2021 (33.3 thousand active cases).

**Figure 2:**
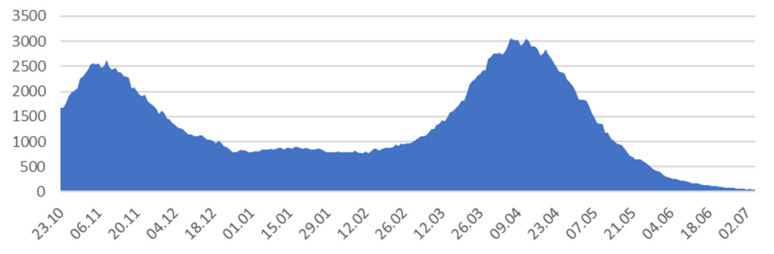
Number of people hospitalized in Małopolska. Source: Data collected on the basis of reports provided by the Ministry of Health, data from the WSEZ, PSSE, Voivodship Offices, and data obtained via requests for access to public information.

The largest number of people hospitalized (Figure 2) during the first wave was on 9 November 2020 (2623 hospitalizations) and during the second wave was on 6 April (3051 hospitalizations).

**Figure 3:**
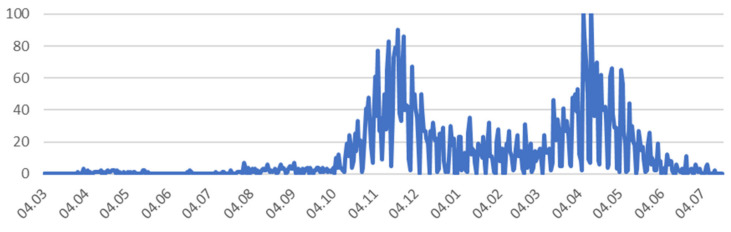
Number of deaths (COVID-19) in Małopolska. Source: Data collected on the basis of reports provided by the Ministry of Health, data from the WSEZ, PSSE, Voivodship Offices, and those obtained in requests for access to public information.

The largest number of deaths (COVID-19) during the first wave was on 11 November 2020 (90 deaths) and during the second wave was on 13 April (110 deaths). At present (22 July 2021) data show a significant reduction in the number of newly diagnosed cases (Figure 3).

## 4. Investment and Organization of the Ward

Żeromski Specialist Hospital in Kraków is a public medical entity that was actively involved in preventing the spread of the virus and treating patients suffering from COVID-19 from the beginning of the SARS-CoV-2 epidemic. Regional governance obliged the hospital to organize beds for patients with COVID-19; at the beginning of the epidemic the number of beds for infected patients was 42, whereas with its development the number increased to 70. Due to the rapid increase in the number of cases, at the end of October 2020 the hospital was required to immediately increase the number of beds, including an increase in the number of ventilator beds.

**Figure 4:**
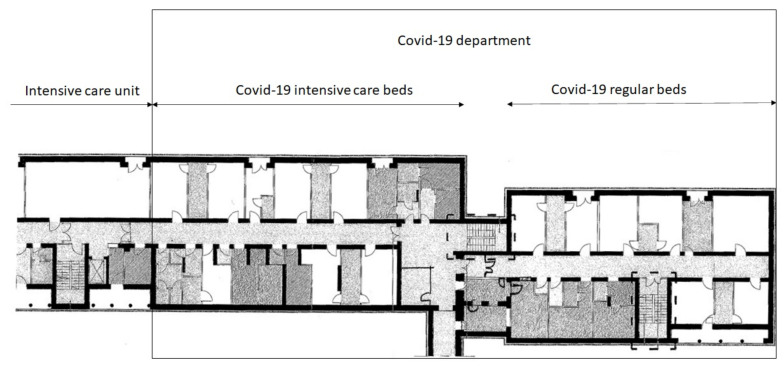
Architectural floor in the hospital source: Hospital internal data.

Due to the lack of sufficient infrastructure in existing Infectious and Internal Departments as well as in the Emergency Department, it was decided to use the rooms of the Surgery Department. This ward is organized into two sections. One section was adjacent to the Intensive Care ward, which was an opportunity to expand the number of ventilator beds for the most seriously ill. After the transformation, a COVID-19 Department was established on the premises of the existing Surgery Department with the potential for:

- 27 general COVID-19 beds,
- 13 COVID-19 intensive care beds (see Figure 4).

The possibility of extracting additional infrastructure required the transfer of the Surgery Department to the beds previously occupied by the Orthopedic Surgery Department and the reduction of the activities of both departments by half. The decision influenced access to surgical and orthopedic services, which is presented below in the financial impact analysis. The intensified demand for hospital beds resulting from patients with COVID-19 generated opportunity costs for patients who could not be admitted to Surgery or Orthopedic Surgery Departments because beds were not available. These opportunity costs were visible not only on the hospital level discussed further but also on the social level. This problem has also been discussed by [19]. 

The organization of the COVID-19 Department required undertaking organizational and investment works aimed at providing the ward with appropriate infrastructure and outlining procedures to ensure the safety of patients and staff.

## 5. Construction Works

Due to the fact that previously there was a surgery ward in the adapted area, the necessary medical infrastructure was available. The specifications of the COVID-19 Department made it necessary to carry out some necessary construction works. Sterile and non-sterile communication paths were separated, partition walls were built to contain the spread of the virus, some of the existing passages were bricked and new ones were created to arrange adequate locks for personnel. The construction works were carried out by technical staff employed at the hospital within working hours. Due to the small scope of construction works and the short deadline for their implementation, the team used building materials left over from previous works, stored at the hospital warehouse. The whole process was completed within six days of the decision on the necessity to establish a new department.

The new beds were equipped with the necessary equipment and oxygen. The equipment was partly borrowed from other departments, and some new purchases were made. They were financed from central subsidies allocated to the fight against COVID-19, as well as financed from the hospital’s own funds.

## 6. Personnel

The medical team of the COVID-19 Department was made up of specialists and residents from various departments. Physicians working in the new department were transferred from other areas of the hospital: infectious diseases, pediatrics, ophthalmology, internal and orthopedics departments. During the day, there were two residents and four specialists in the ward. Two residents and one specialist were on duty at night.

The nursing team was composed of nurses transferred from other departments as well, specifically from orthopedic, surgical, otolaryngology, gynecology and obstetrics, dermatology, pediatrics and infectious disease departments. On average, there were four nurses per day shift and four per night shift under the 12-h system. Nurses were changed in the non-sterile area approximately every 4 h. There was additionally a medical assistant and a medical secretary on working days on the day shift.

Due to the different parent departments of the medical staff, managing the newly established COVID-19 Department was a great challenge. The department management consisted of a head physician and a head nurse. The head physician was an infectious disease specialist, previously working in the Infectious Department. The head nurse was an experienced head nurse who was delegated to the Infectious Department during the first wave to gain management experience during the pandemic. The experience of the department’s managers made it possible to improve the organization of medical personnel with diverse work experience. 

## 7. Triage of the Patients

**Figure 5:**
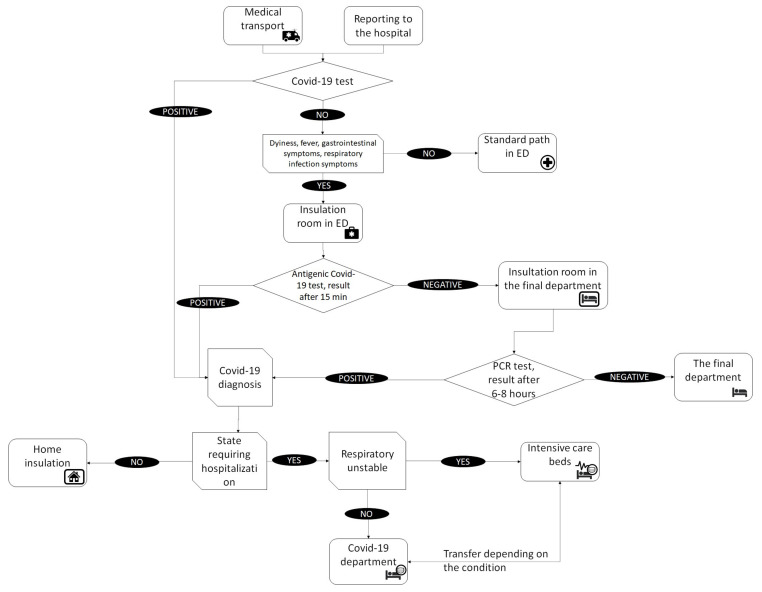
Triage procedure source: Hospital internal data.

Patients with suspected COVID-19 were admitted to the hospital through the hospital emergency department in accordance with the adopted triage procedure (see Figure 5). Patients were transported by medical transport ambulances or came individually. Based on the preliminary interview, the special triage route covered patients with a positive COVID-19 test result, those with dyspnea, fever, and gastrointestinal symptoms, and those with symptoms of respiratory infection.

Patients who had a positive COVID-19 test result on admission to hospital were either admitted to the hospital or sent to COVID-19 isolation centers or home isolation, depending on their condition. Further treatment in the hospital took place in infectious disease departments, among others the COVID-19 Department.

In the case of symptomatic patients without a test result, the first stage of the procedure took place in the Emergency Department, in which an interview was conducted and an antigen test performed. During the examination and while waiting for the result, the patient was kept in an isolation room. The test result was obtained after 15 min. In the case of a positive result, the patient fell into the same path as patients positive at admission. Their further treatment depended on the condition. If the antigen test was negative, a PCR test was conducted for the presence of SARS-CoV-2 virus. While waiting for the test result, the patients stayed in an isolation room either in the ED or in the target department, especially if they required immediate specialist treatment. After receiving the PCR test result, the patient either remained in the target department in a general room if the result was negative and the condition required it, or went to the infectious department.

In the infectious department, oxygen-unstable patients went to intensive care beds equipped with oxygen therapy devices, while the others stayed in general beds. The condition of patients was constantly monitored by medical staff and in the event of deterioration or improvement, patients were transferred in order to provide the best possible care to patients in the most serious condition.

## 8. Safety of Staff and Patients

The hospital developed a number of procedures for dealing with infectious patients, in particular those infected with SARS-CoV-2. Their creation was the responsibility of the medical director, acting in this area in cooperation with the head of the Epidemiology Department. The procedures concerned the determination of communication paths and hospitalization of sick patients, including their isolation from other patients, while at the same time prohibiting the possibility of visiting the ward while an infected person was there.

An important procedure involved determining the rules of infection prevention obligatory in all areas of the hospital. In medical units, the staff was trained in the procedures applicable to the specific area, including in particular:

- type of personal protective equipment used, specified based on the conditions, personnel and scope of activities undertaken;
- collecting biological material from the patient;
- maintaining the hygiene of the hospital environment.

Basic personal protective equipment included barrier aprons with long sleeves, caps, disposable gloves (in the amount of two pairs put on top of each other), masks (surgical or protective type FFP2/FFP3 for aerosol-generating procedures), protective glasses or a protective helmet.

**Figure 6:**
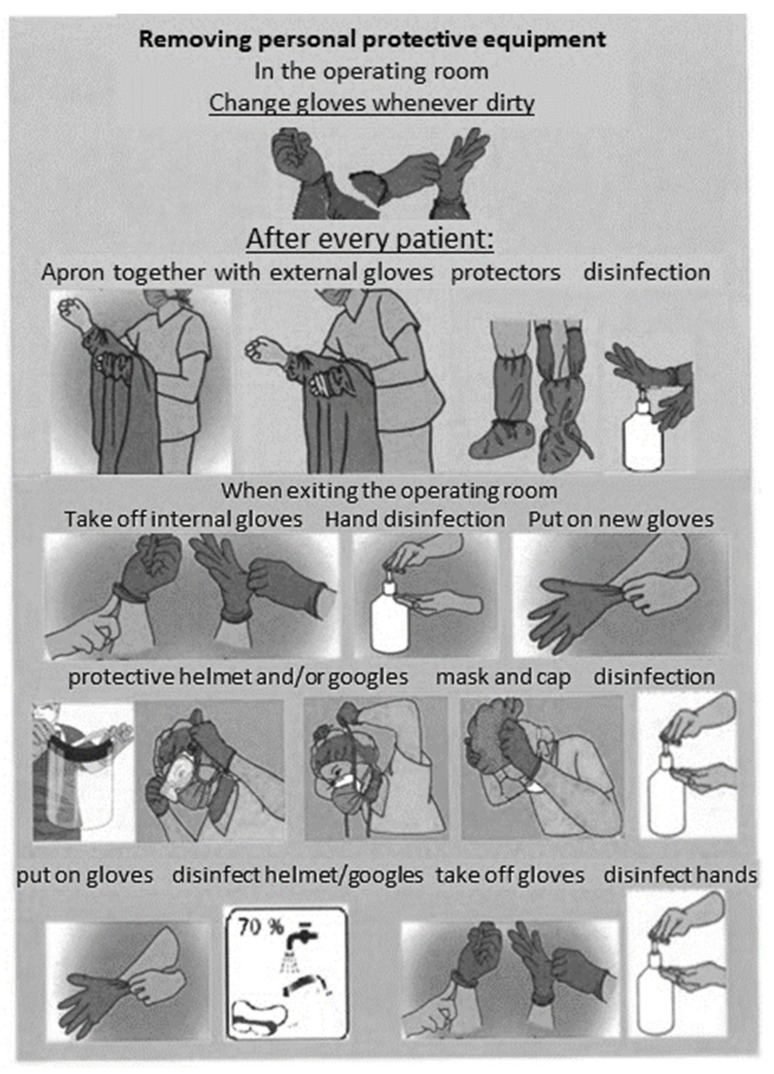
Infographics about using personal protective equipment. Source: Hospital internal data.

In addition to providing personnel with personal protective equipment, employees were trained in how to wear it, and given precise determination of its maximum duration of use. The standard of conduct was defined separately for various organizational units, including the most sensitive ones such as the operating theater, intensive care unit or treatment rooms. In the places where staff dressed, colored infographics were installed showing the correct way to put the equipment on (see Figure 6).

When biological material was collected, the patient had to, if possible, be equipped with a mask, and the staff must have worn personal protective equipment, which must afterwards have been removed in a designated area in a specific order and placed in appropriate medical waste bags. If the collection of the material required patient transportation, all transportation routes and places where the patient was present had to be disinfected with virucidal agents immediately afterwards.

## 9. Functioning of the COVID-19 Department

The department operated for two months from 23 October to 31 December. After this period, due to the reduction in the number of cases and the decrease in the strength of the second wave of the epidemic, the patients were transferred to other departments of the hospital, and the rooms of the COVID-19 Department were rearranged back into the Surgery Department. 

During the two-month period of operation, 380 patients were admitted to the COVID-19 Department, including 280 patients in COVID-19 intensive care beds. Eighty-five patients were discharged from the hospital due to the stabilization of their condition, while another 160 patients were transferred within the hospital to another department.

The average length of stay of patients varied depending on their condition. Patients in the general COVID-19 beds stayed on average 11 days, while the average duration of stay in COVID-19 intensive care beds was 14 days. The total number of deaths during the analyzed period was 130, with the percentage of deaths significantly different depending on the section. In the general section, the death rate was 30%, while in the intensive care section, it was 36%, which significantly exceeded the average mortality rate in the Intensive Care Unit before the pandemic (22% in 2019).

In the third wave, which began in February 2021, the COVID-19 Department started operating in the same location again.

## 10. Consequences for Hospital’s Finances

The 40-bed COVID-19 Department received per-diem financing from public funds from the moment it was opened. The rate per diem depended on the patient’s condition (measured by the level of oxygen saturation). For saturation below 95, the nightly rate was 166 USD, and for a higher saturation, 87 USD. In addition, the hospital received a lump sum payment in the event of keeping empty beds in the ward ready to admit patients. The database of available beds in all hospitals in the region was used by emergency medical teams when patients were transported to the nearest hospital with available beds.

The establishment and operation of the branch was associated with the following costs:

- equipping the ward with the necessary medical equipment;
- personal protective equipment;
- variable costs of treatment;
- medicines and medical materials;
- diagnostic services;
- other costs of stay;
- salaries of medical personnel.

The salary costs were treated as incremental even though the personnel were transferred from other departments of the hospital. Firstly, wages on the COVID-19 Department were partly paid for overtime work. Secondly, the cost of wages in other departments was not reduced, as either other personnel were hired or the remaining workers were paid overtime. The only exceptions were the Surgery and Orthopedic Surgery Departments in which capacity was reduced. This has been included in the analysis. 

The COVID-19 Department was established in the rooms of the Surgery Department, which was moved to the area occupied by the Orthopedic Surgery Department. As a result of this decision, the availability of both departments was significantly limited. In both departments, some services are financed FFS, which resulted in a loss in their income.

Table 1 summarizes the incomes and direct costs of the COVID-19 Department broken down into three categories, the lost revenue of the Surgery and Orthopedic Surgery Departments, and the savings on variable costs in these departments due to the lower number of patients as well as on the personnel costs moved to the COVID-19 Department.

The financial consequences included both the revenues of the department and the related direct costs, as well as benefits lost. Direct costs are divided into three basic groups:

- costs of adjusting the ward, equipment and personal protective equipment;
- variable costs of treatment;
- salaries of medical personnel.

Fixed costs of the Surgery and Orthopedic Surgery Departments did not change and were not included in the analysis. The only exception was savings on personnel salaries, which resulted from the smaller number of people on duty in these departments. In the Surgery Department it was the full-time equivalent of one nurse position and in the Orthopedic Surgery Department the equivalent of two nurse positions. The reduction in nursing staff costs was disproportionately lower than the reduction in the scale of operations, due to applicable regulations and the fact that many staff were sent home to quarantine or took sick leave with pay. No savings on physicians’ costs were generated due to the fact that on-call duty remained unchanged despite the much smaller number of patients in these departments. The number of doctors on call is determined by law and cannot be changed.

The costs of adjusting the ward, equipment and personal protective equipment were incurred as a direct result of the need to organize a new ward and adapt it to the treatment requirements of COVID-19 patients. Due to the fact that work was carried out by the technical staff employed at the hospital and using construction materials left over after previous works (treated as sunk cost), this section only includes incremental costs that would not have been incurred under other conditions.

The variable costs of treatment include medicines and medical and non-medical materials used directly for patients in the COVID-19 Department, as well as the costs of diagnostic services and other costs of stay. 

The personnel salaries include wages for the medical and non-medical personnel of the COVID-19 Department. The analysis takes into account the entire cost of personnel remuneration, while taking into account the savings in the analyzed Surgery and Orthopedic Surgery Departments. No significant savings were recorded in the remaining departments where the medical personnel came from, as staff shortages were compensated for by overtime work of other employees. It is worth noting that such a condition cannot be maintained in the long term due to the high operational risk associated with it. Work in the COVID-19 Department was associated with a special designated remuneration, which was included in the analysis.

## 11. Conclusions

In the face of the second wave of the COVID-19 epidemic, the hospital was obliged to provide medical care and cover the increased demand from infectious patients. The hospital provided care on standard beds and COVID-19 intensive care beds, while continuing its regular activities. The reorganization of the ward took place in a very short time, necessitated by the crisis situation. The overall goal of the reorganization was to increase the capacity needed to treat the increased number of patients with COVID-19 requiring hospitalization. It should be mentioned that this goal was reached in the two-month period; 380 patients were admitted to the COVID-19 Department, including 280 patients on COVID-19 intensive care beds. The aim of the article was to present the organizational and investment steps taken to establish and maintain an infectious hospital ward as well as the consequences of this decision.

Investments made in the hospital were carried out using the existing infrastructure so that they required the least amount of time and ensured the fastest opening of the ward. It is worth noting that the expenses related to the transformation of the department into an infectious disease one were marginal, accounting for less than 1% of the total costs. In a situation where there is underutilized infrastructure, transforming departments into infectious ones is an important opportunity to increase the bed potential, which is an alternative to the construction of temporary hospitals. It is worth noting that in both cases the problem of providing medical personnel to care for patients in these departments remains.

From the point of view of ensuring the continuity of care in the hospital and limiting contact between patients with confirmed COVID-19 and others, the triage and patient qualification system are extremely important. Such procedures were implemented in the hospital, which is consistent with the conclusions of other studies in this area [7,8]. 

Treatment of infectious patients requires the provision of appropriate medical infrastructure and personal protective equipment for the personnel. In the case of the analyzed hospital, mostly preexisting infrastructure was used; new equipment was provided by the central authorities. The hospital’s expenses were mainly related to medical supplies. Expenditure on personal protective equipment and disinfectants accounted for almost 25% of all costs. In addition to providing the equipment, training in its use was conducted in the hospital and relevant infographics were installed. This was consistent with the results of studies showing the importance of proper training of medical personnel [12]. 

The functioning of infectious wards is associated with increased staffing of medical personnel. Additionally, obligatory financial supplements for medical personnel were introduced in Poland during the pandemic. Both of these factors increased the cost of human resources. The analyzed cost structure was dominated by the cost of remuneration, primarily of medical personnel. The Polish health care system lacked human resources, which determined the hospital’s patient care capabilities. In the present analysis, these costs accounted for over 58% of direct costs. An important cost item, resulting from the specificity of treating highly infectious diseases, was patient diagnostics, treatment and costs directly related to the stay (including food) accounting for slightly more than 16%.

In this case, an additional element necessary to include in the analysis is that the limitation of the hospital’s potential due to the reorganization of the existing departments was associated with limiting access to care provided therein. In the discussed case, these were the surgical and orthopedical services.

To estimate the level of lost benefits, the average rates of FFS settled revenues per person-day were taken into account, as well as variable costs that were not incurred due to the lack of admission of patients. While it was assumed that some of these patients would be admitted in the future, the hospital would not be able to fully make up for the ward downtime resulting from less availability. Both revenues and costs were estimated in relation to the level of average values for the previous year (i.e., 2019). Variable costs in the surgery and orthopedics departments accounted for 38.7% and 39.3% of revenues, respectively.

When planning organizational changes in hospitals during a pandemic, decision-makers should take into account the full financial consequences of this action, which are wider than those resulting from direct costs (resulting from additional employment or medical materials). Hospitals receive fixed budgets for the majority of services provided in the wards, but some benefits are charged on the basis of fee for service. Therefore, a change in hospital organization due to an epidemic not only results in costs associated with personnel and medical supplies, but may also lead to lost income. 

All in all, the financial losses of the hospital, not reflected in its revenues, amounted to almost 0.5 million USD within two months. Such a loss might be difficult to be recovered from the hospital’s regular activity and may worsen the hospital’s financial standing in the long term. If such a case occurred for the majority of hospitals involved in treating patients in pandemics, that would reflect an asymmetric transfer of the pandemic-related risk from the payers to the healthcare provider.

This study has several limitations. There are some costs resulting from an epidemic that were difficult to capture and have been excluded from this analysis. Some COVID-19 patients were admitted to wards other than the infectious departments ward due to the need for surgical treatment. However, COVID-19 Department personnel participated in the process of their treatment. Due to the lack of data allowing the identification of these cases, they were excluded from the analysis.

Further, taking into account the lost benefits in the analysis, we assumed that the efficiency of the Surgery and Orthopedic Surgery Departments would not increase significantly, which would not make it possible to make up for the losses resulting from the epidemic in a later period. We believe that this assumption is correct due to the lack of premises allowing expectations of a sudden improvement in efficiency. On the other hand, we were not sure that patients would necessarily appear in the hospital in autumn 2020 even if the availability of the Surgery and Orthopedic Surgery Departments was not limited. This could be influenced by other factors, such as patients’ fear related to the epidemic or their health conditions making it impossible to carry out scheduled procedures. However, it is difficult to estimate the influence of these factors due to the lack of a comparative period with similar environmental conditions. It was therefore decided to omit this element due to the fact that there are long waiting lists for both departments.

## Figures and Tables

**Table 1:** Analysis of the financial consequences of the organization of the COVID-19 Department.

		in USD
**A**	**Revenues**	**1,886,162**
I	per diem payment for COVID-19 patients	561,890
II	lump sum payment for epidemic readiness	1,324,272
**B**	**Direct costs**	**1,415,729**
I	Costs of adjusting the ward, equipment and personal protective equipment	**358,062**
I.a	medical equipment	7700
I.b	personal protective equipment	338,748
I.c	disinfectants	11,615
II	Variable costs of treatment	**228,252**
II.a	medicines and medical materials	155,235
II.b	diagnostic services	30,796
II.c	other costs of stay	42,221
III	Personnel salaries	**829,415**
**C**	**Benefits lost**	**936,789**
I	Surgery Department	153,027
I.a	Revenues	249,636
I.b	Variable costs not incurred	96,609
I.c	Personnel salaries not incurred	54,907
II	Orthopedic Surgery Department	783,762
II.a	Revenues	1,291,206
II.b	Variable costs not incurred	507,444
II.c	Personnel salaries not incurred	109,815
**D**	**Result (A-B-C)**	**−466,356**

## Data Availability

The statistics on coronavirus infections included in this article can be accessed through the website: https://www.gov.pl/web/koronawirus/wykaz-zarazen-koronawirusem-sars-cov-2, accessed on 22 July 2021.
